# Apm4, the mu subunit of yeast AP-2 interacts with Pkc1, and mutation of the Pkc1 consensus phosphorylation site Thr176 inhibits AP-2 recruitment to endocytic sites

**DOI:** 10.4161/cib.28522

**Published:** 2014-04-02

**Authors:** Bernardo Chapa-y-Lazo, Kathryn R Ayscough

**Affiliations:** Department of Biomedical Science; University of Sheffield; Sheffield, UK

**Keywords:** protein kinase C, endocytosis, *Saccharomyces cerevisiae*, adaptor proteins, phosphorylation

## Abstract

The AP-2 endocytic adaptor has been extensively characterized in mammalian cells and is considered to play a role both in cargo binding and in formation of endocytic sites. However, despite our detailed knowledge of mechanistic aspects of endocytic complex assembly and disassembly in the model organism *Saccharomyces cerevisiae*, no function of AP-2 had been described in wild-type yeast under normal growth conditions. A recent study however revealed that disruption of the complex caused by deletion of the gene encoding its mu subunit (*APM4*) caused defects in cell polarity such that responses to pheromone, nutritional status and cell wall damage were affected. Furthermore, a homozygous deletion of the mu subunit gene in *Candida albicans* affected its ability to grow hyphae. Direct binding to the yeast cell wall stress sensor Mid2 was detected, and in an *apm4* deletion strain Mid2 showed reduced re-localization to the mother bud neck region following cell wall damage with calcofluor or to the mating projection tip. Here we demonstrate an interaction between Apm4 and the yeast cell wall integrity pathway component Pkc1 and show that mutation of the predicted Pkc1 site in the Apm4 hinge region affects recruitment of the AP-2 complex to endocytic sites.

The endocytic adaptor AP-2 links plasma membrane bound cargoes to the clathrin coat of forming vesicles. It is a hetero-tetramer in which two large subunits, α and β form the core of the module as well as interacting with clathrin and other endocytic coat proteins, while its mu and sigma subunits interact with the core and are also able to bind cargo through defined motifs.^1^^,^^2^ All 4 subunits are present in *S.cerevisiae* but deletion of any of the components does not compromise the formation, or invagination of endocytic sites.^3^^,^^4^ Deletion does however correlate with resistance to the killer toxin K28.^3^ These observations led to the idea that AP-2 in yeast might function as a cargo binding adaptor but does not play a mechanistic role in endocytosis.

In mammalian cells regulation of AP-2 cargo binding is proposed to occur by phosphorylation of the Thr156 residue which lies in the hinge region between the N-terminal longin and C-terminal mu adaptin homology domain.^5^^-^^7^ Mutation of this site to a non-phosphorylatable residue inhibits cargo uptake, and it is suggested that the modification might stabilize the ‘open’ conformation of the AP-2 complex to enable cargo interaction at the membrane. Phosphoproteome analysis of *S.cerevisiae* cells has led to the identification of a heavily phosphorylated region in AP-2 (between Ser157 and Ser 188) corresponding to the hinge region in mammalian mu suggesting that like its mammalian counterpart, yeast AP-2 might be regulated by phosphorylation to ensure cargo binding is spatially and temporally controlled.

A yeast two hybrid screen was conducted commercially (Hybrigenics) with full-length Apm4 (residues 2–491) in both LexA based and Gal4 based assays. This led to the identification of 9 interactors with a good level of confidence, with at least two distinct plasmids isolated in each case. These are listed in Table 1. Importantly, a clear interaction was found with the large AP-2 β adaptin subunit Apl1, which has previously been found to interact with the Apm4 mu subunit.^4^ The largest number of hits was found with the yeast protein kinase C Pkc1, and interactions were found with both libraries. All 11 distinct sequences isolated overlapped to give a minimum interaction site in Pkc1 of 408–565 that corresponds to the C1 domains in Pkc1 (Fig. 1A). This region has previously been shown to be required for interaction of Pkc1 with Rho1 a small GTPase required for localization of Pkc1 to the bud neck and tip^8^^-^^10^ raising the possibility that AP-2 could be involved in this localization. In support of this Pkc1 localization was found to be disrupted in the *apm4* deletion strain.^11^ Given that both Rho1 and Apm4 appear to interact with the same C1 domain region of Pkc1 it could be envisaged that Pkc1 binds AP-2 complex thus allowing it to be constantly re-polarized to growing sites. At sites of cell wall stress, when Rho1 is active, this small GTPase may bind to Pkc1 competitively, and release it from AP-2-mediated re-internalisation thus allowing active Pkc1 to be localized and maintained at sites of stress. Inactivation of Rho1 could in turn allow re-association of AP-2 with Pkc1 and its recycling.

**Table 1.** 

Gene name	Hits (distinct)	Smallest Fragment (amino acid)	Function
*PKC1/YBL105c*	30(11)	410–531	Cell Wall integrity kinase
*APL1/YJR005w*	11(4)	178–459	AP-2 β subunit
*NCE103/YNL036w*	9(6)	42–221	Carbonic anhydrase
*RAD18/YCR066w*	7(3)	66–155	E3 ubiquitin ligase
*GEA1/YJR031w*	7(6)	1240–1408	Guanine nucleotide exchange factor for ADP ribosylation factors involved in vesicular transport between Golgi and ER, Golgi organization and actin cytoskeleton organization
*FUN12/YAL035w*	2(2)	592–906	Translation initiation factor eIF5B
*HOM3/YER052c*	3(2)	1–55	Aspartate kinase
*DMA1/YHR115c*	2(2)	82–301	E3 Ubiquitin-protein ligase; controls septin dynamics and regulates recruitment of Elm1p to bud neck
*ATG11/YPR049c*	2(2)	827–1109	Adaptor protein for pexophagy and the Cvt targeting pathway

**Figure:**
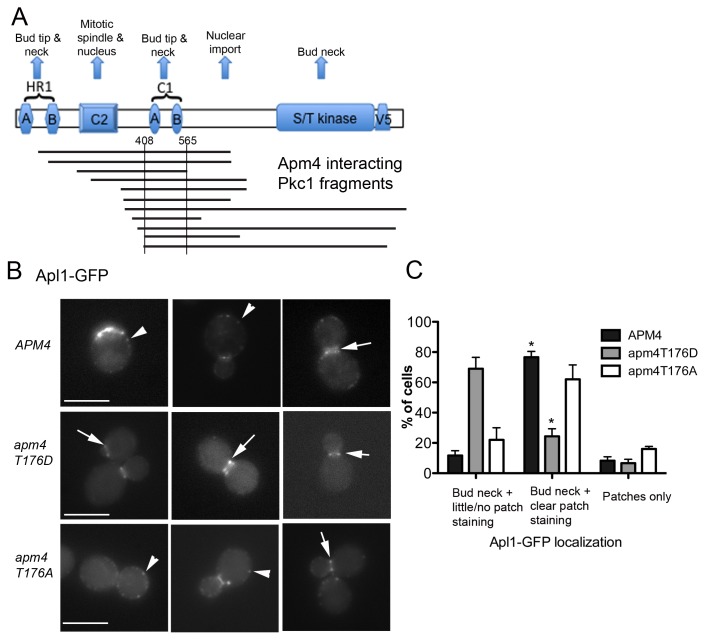
**Figure 1.** . Apm4 interacts with Pkc1. (A) A yeast two hybrid screen with Apm4 as bait identified 11 different interacting plasmid carrying segments of Pkc1. The minimal overlapping region of these is marked with vertical lines. Recognized domains of Pkc1 are marked, and the roles attributed to these domains from genetic studies are indicated (B) KAY1747 cells (*MAT****a****his3∆1, leu2∆, ura3∆, met15∆, APL1-GFP::HIS3 apm4∆::KanMx)* were transformed with plasmids expressing wild type, T176A or T176D mutants. Cells were grown to log phase and imaged using an Olympus IX-81 inverted microscope with a Photometrics Cool Snap HQ2 cooled CCD camera, and Image ProPlus image capture software. Arrowheads indicate localization to endocytic patches; arrows show mother-bud neck localizaton. Shown are representative images of cells at different stage of the growth cycle. Bar = 5 µm. (C) Budding cells were analyzed (n ≥ 30 cells in n = 3 experiments). Localization of Apl1-GFP was assessed as being at the bud neck or in patches. Some cells showed only very weak patch staining with localization just visible above cytosolic background. These were classified as ’bud neck with little/no patch staining’. A 2 way Anova test indicated that there was a significant reduction (p value ≤ 0.0001; marked *) in cells showing bud neck and clear patch staining in the T176D mutant compared with wild type. Error bars are standard deviation.

The sequence of Apm4 was analyzed using NetPhosK^12^ to identify the amino acids most closely fitting a Pkc1 phosphorylation site. The only site with probability ≥ 0.9 was at T176 which lies in the hinge region between the N- and the C-terminal domains of Apm4. T176 was mutagenised to alanine (non-phosphorylatable) or to aspartate (phosphomimetic) forms using a QuikChange Lightning kit (Stratagene). As shown (Figs. 1B,C), plasmid borne *APM4* is able to restore normal Apl1-GFP (β adaptin) localization in *apm4*∆ cells in which deletion of *apm4* was previously shown to cause loss of Apl1-GFP localization.^11^ Both the Apm4T176A and Apm4T176D mutants were able to restore localization to the neck region of cells, but there was a significant reduction in the proportion of cells expressing the T176D mutant that localized Apl1-GFP to cortical patches (76 ± 4% wild type cells have bud neck staining with clear endocytic patches compared with only 24 ± 5% in cells expressing *apm4*T176D mutant). This result suggests that phosphorylated Apm4 may function to recognize and internalise material to be relocated to the neck region but unless subsequently dephosphorylated may not reassociate at endocytic sites.

To conclude, these data add to those previously published^11^ and indicate that like AP-2 of mammalian cells, the yeast AP-2 mu can interact with Pkc1 and that this interaction modulates the function of the adaptor in endocytosis.
